# Supplementary material for: Identification of photoperiod-induced specific miRNAs in the adrenal glands of Sunite sheep (Ovis aries)
Source: Front Vet Sci. 2022 Jul 22;9:888207. doi: 10.3389/fvets.2022.888207 (PMC9354845; doi:10.3389/fvets.2022.888207)
Supplement: Supplementary file 1 [file Data_Sheet_1.ZIP › Supplementary file/miRDeep2 file.zip!/miRDeep2 file/Know_Structure/SPLP42A1/oar-mir-152.pdf]

miRBase precursor : oar-mir-152
Total read count : 19908
oar-miR-152 read count : 19908
remaining reads : 0

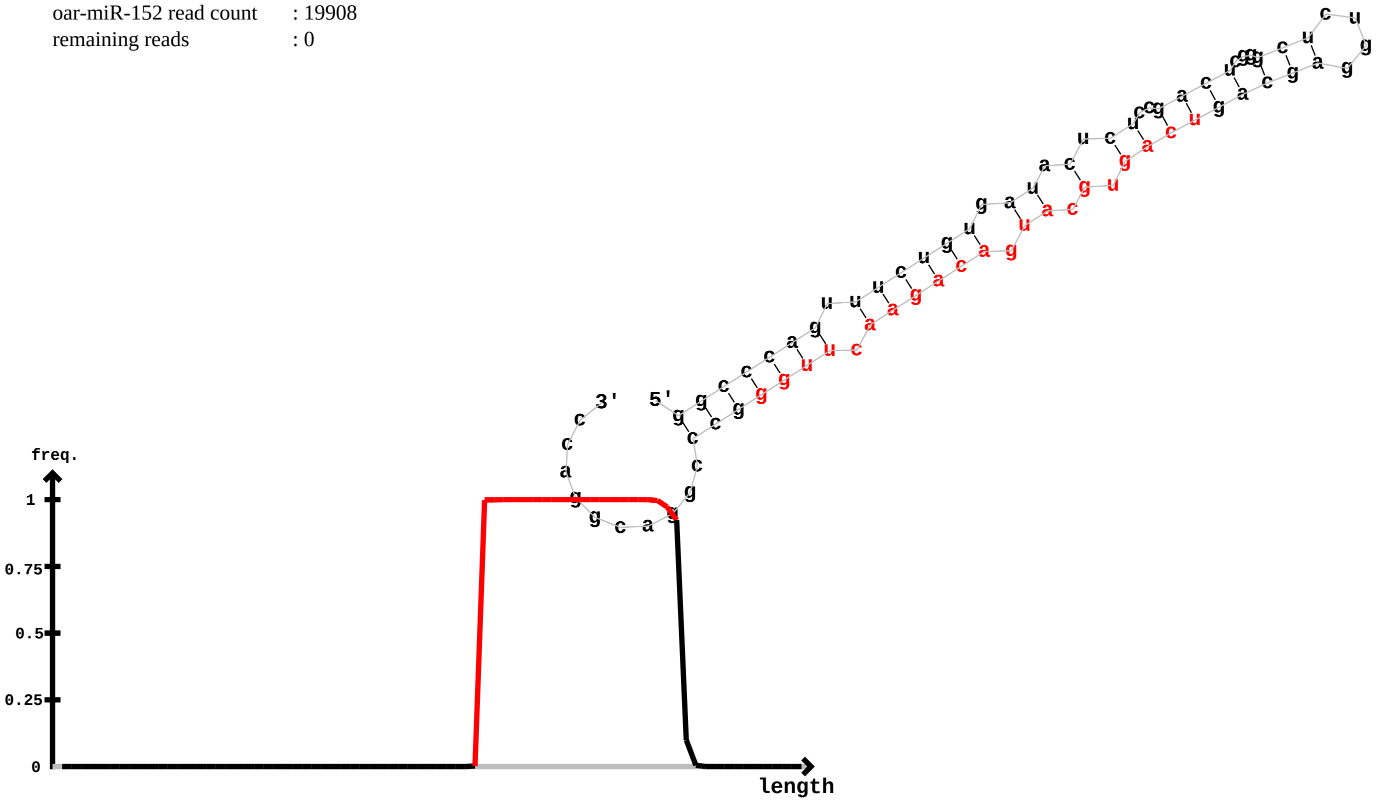


```
                                                   oar-miR-152
5'- ggcccaguuucugugauacucuccgacucgggcucuggagcagucagugcaugacagaacuugggcccggacggacc   -3'    exp
    (((((((.((((((.((.(.((..((((...(((....))))))))).).)).)))))).))))))).........     reads      mm         sample
    ..........................................gucagugcaugacagaacuu...............     1          0          seq
    ..........................................gucagugcaugacagaacuug..............     9          0          seq
    ..........................................gucagugcaugacagaacuugg.............     29         0          seq
    ..........................................gucagugcaugacagaacuuggg............     2          0          seq
    ...........................................ucagugcaugacagaac.................     2          0          seq
    ...........................................ucagugcauCacagaac.................     1          1          seq
    ...........................................ucagugcaugacagaacu................     45         0          seq
    ...........................................ucagugcauCacagaacu................     12         1          seq
    ...........................................ucagugcaugacagaacuu...............     447        0          seq
    ...........................................ucagugcauCacagaacuu...............     27         1          seq
    ...........................................ucagugcaugacagaacuug..............     970        0          seq
    ...........................................ucagugAaugacagaacuug..............     2          1          seq
    ...........................................ucagugcaugacagaacuugg.............     16364      0          seq
    ...........................................ucagugcaugacagaacuuggg............     1880       0          seq
    ...........................................ucagugcaugacagaacuugggc...........     94         0          seq
    ...........................................ucagugcaugacagaacuugggcc..........     1          0          seq
    ............................................cagugcaugacagaacuugg.............     7          0          seq
    .............................................agugcaugacagaacuu...............     1          0          seq
    .............................................agugcaugacagaacuug..............     1          0          seq
    .............................................agugcaugacagaacuugg.............     9          0          seq
    .............................................agugcaugacagaacuugA.............     1          1          seq
    .............................................agugcaugacagaacuuggg............     2          0          seq
    ..............................................gugcaugacagaacuuggA............     1          1          seq
```
